# Persistent Esophageal Parakeratosis With Recurrent Candida Esophagitis in a Virally Suppressed Person With HIV

**DOI:** 10.7759/cureus.98257

**Published:** 2025-12-01

**Authors:** Rizgar Hama-Salih, Mohammad Alsaadony, Kushal Panja, Deepa Bai, Madiha Erashdi, Emil Salmo

**Affiliations:** 1 Gastroenterology, Tameside Hospital, Tameside and Glossop Integrated Care NHS Foundation Trust, Manchester, GBR; 2 General Internal Medicine, North Manchester General Hospital, Manchester University NHS Foundation Trust, Manchester, GBR; 3 General Medicine, North Manchester General Hospital, Manchester University NHS Foundation Trust, Manchester, GBR; 4 General Medicine, Manchester University NHS Foundation Trust, Manchester, GBR; 5 Pathology, Liverpool University Hospital NHS Foundation Trust, Liverpool, GBR; 6 Pathology, Royal Oldham Hospital, Northern Care Alliance NHS Foundation Trust, Manchester, GBR

**Keywords:** candida esophagitis, dysphagia, esophageal parakeratosis, esophagogastroduodenoscopy, hiv, ogd

## Abstract

Esophageal parakeratosis is an uncommon histopathologic finding that can closely mimic malignancy endoscopically and has been linked to micronutrient deficiency states and chronic reflux. We report a 57-year-old man with well-controlled HIV (stable viral suppression and robust CD4 count) who developed progressive dysphagia with malignant-appearing esophageal plaques over a long segment of the mid-to-distal esophagus. Serial biopsies repeatedly showed parakeratosis/hyperkeratosis without dysplasia or malignancy, with intermittent Candida on histology. Despite the alarming appearance, cross-sectional imaging revealed no mass or nodal disease. Management included high-dose acid suppression, short courses of fluconazole for Candida esophagitis, and empiric zinc and riboflavin supplementation; the endoscopic extent of keratinization reduced but persisted, and dysphagia fluctuated without a fixed stricture. Given ongoing symptoms and easy passage of the scope, esophageal manometry was arranged to evaluate a motility contribution, and surveillance endoscopy was planned. This case emphasizes the importance of repeat histology before committing to oncologic pathways, consideration of micronutrient supplementation, cautious use of long-term proton-pump inhibition in patients at risk for Candida, and individualized surveillance where formal guidance is lacking.

## Introduction

Esophageal parakeratosis is a rare finding characterized histologically by a hyperkeratinized squamous epithelium with retained pyknotic nuclei, often presenting endoscopically as white plaques or thickened mucosa that can closely mimic dysplasia or early neoplasia [1]. In the limited case literature, some reports have linked its development to micronutrient deficiencies, particularly riboflavin and zinc, and observed partial regression after repletion [2,3].

Candida esophagitis (CE), classically associated with immunocompromise, is increasingly recognized among patients without profound immunosuppression. In a cohort of “immunocompetent” patients, proton-pump inhibitor (PPI) use was identified as the most common associated risk factor (odds ratio (OR) ~1.69) [4]. A larger Japanese cross-sectional study of 7,736 patients undergoing endoscopy found PPI use (OR 1.69), diabetes, atrophic gastritis, and gastrectomy to be independent risk factors for CE even in patients without overt immunodeficiency [5]. In a clinical series, 63-81% of esophageal candidiasis cases had coexisting exposure to PPIs, underlining the strong pharmacologic association [6]. These data suggest that acid suppression may predispose to fungal overgrowth via hypochlorhydria and local mucosal changes.

Given the striking endoscopic mimicry of neoplasia in parakeratosis, the paucity of surveillance guidelines, and the potential overlap with CE, even in adequately immunocompetent or virologically suppressed hosts, this case offers an opportunity to refine practical strategies in diagnosis, management, and follow-up.

## Case presentation

A 57-year-old man with HIV-1 diagnosed in 2008 (consistently virologically suppressed) had longstanding reflux symptoms beginning in 2016. He also had intermittent episodes of esophageal candidiasis over the years and comorbidities, including cleared hepatitis B infection, gastroesophageal reflux disease, irritable bowel syndrome, steatotic liver disease with a normal FibroScan in 2017, psoriasis, chronic obstructive pulmonary disease (COPD)/asthma, and hypertension.

The patient was not receiving systemic corticosteroids, inhaled corticosteroids, biologic agents, or any form of immunosuppressive therapy. His COPD/asthma was managed only with an as-needed salbutamol inhaler. His psoriasis was mild and well-controlled without systemic treatment. His antiretroviral therapy consisted of a non-boosted regimen (tenofovir disoproxil fumarate, emtricitabine, and doravirine).

In August 2019, an esophagogastroduodenoscopy (OGD) performed for symptoms of dysphagia showed esophagitis. In February 2022, a repeat OGD revealed a white patch in the mid esophagus; biopsies confirmed candidiasis, and a 14-day course of fluconazole led to symptomatic improvement.

**Figure 1:**
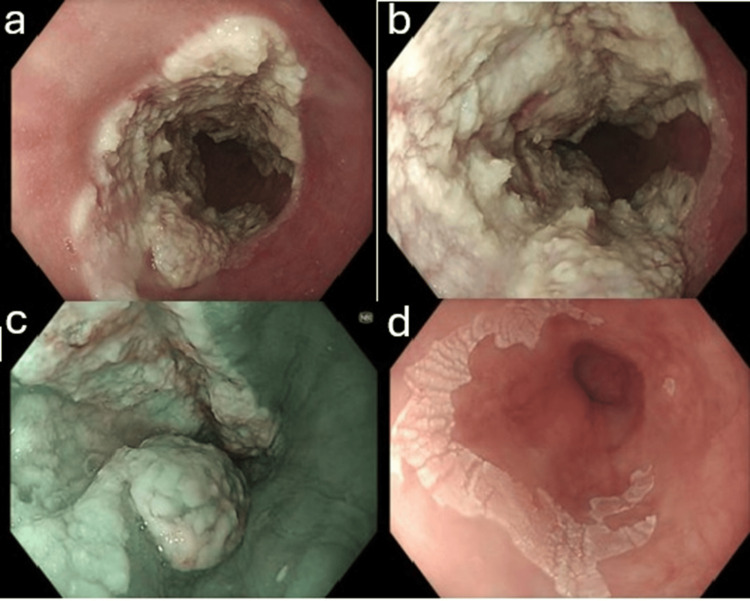
OGD showing a malignant-appearing, exophytic, passable lesion with severe grade D esophagitis, with invasive esophageal candidiasis in the mid esophagus from 31 cm to 39 cm, and a separate superficial patch at 41 cm. (a and b) Severe grade D esophagitis and possible invasive esophageal candidiasis. (c) Malignant-appearing exophytic lesion. (d) Superficial patch at 41 cm. OGD, esophagogastroduodenoscopy

In May 2024, he developed a recurrence of progressive dysphagia with dyspepsia and weight loss. An OGD was performed, which showed malignant-appearing, severe grade D esophagitis spanning 31-39 cm from the incisors, with a separate superficial patch at 41 cm (Figure 1).

Histology showed signs of candidal infection and bacterial clusters. No dysplasia or malignancy. A computerized tomography (CT) scan of the thorax/abdomen/pelvis in May 2024 demonstrated no mass, no mediastinal or abdominal lymphadenopathy, and no sinister lesions; notably, the endoscopic lesion was not appreciable on CT. At the radiology and upper gastrointestinal (GI) multidisciplinary team (MDT) meeting later that month, high-dose PPI, a 14-day fluconazole course, empiric *Helicobacter pylori* eradication, and a repeat OGD were recommended.

**Figure 2:**
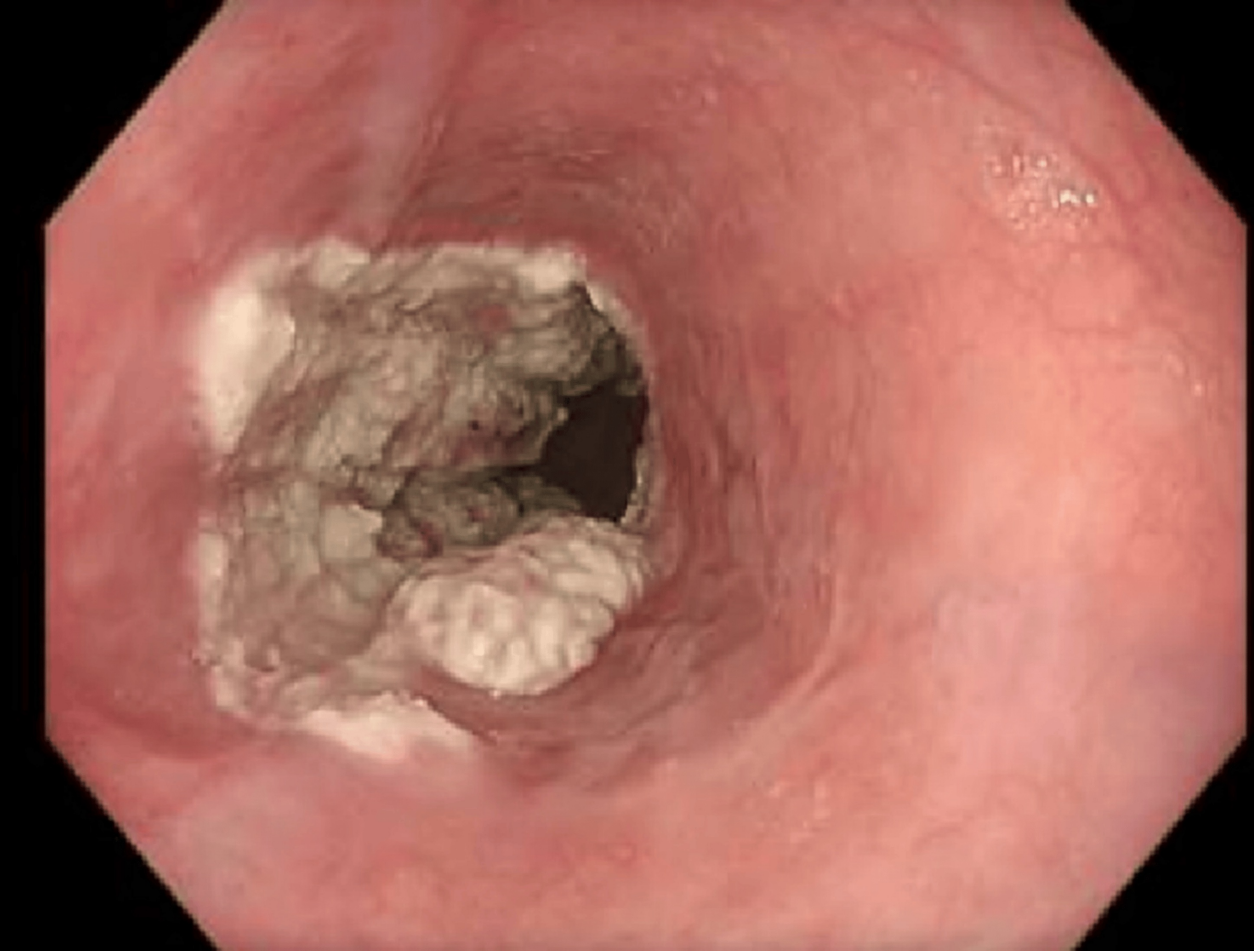
OGD showing a keratectatic/fungoid 5-cm lesion at approximately 30 cm. OGD, esophagogastroduodenoscopy

A repeat OGD in July 2024 described a “keratectatic fungoid” 5-cm lesion at ~30 cm. The differential included esophageal papillomatosis versus keratosis (Figure 2). Histopathology ruled out papillomatosis and confirmed keratosis.

**Figure 3:**
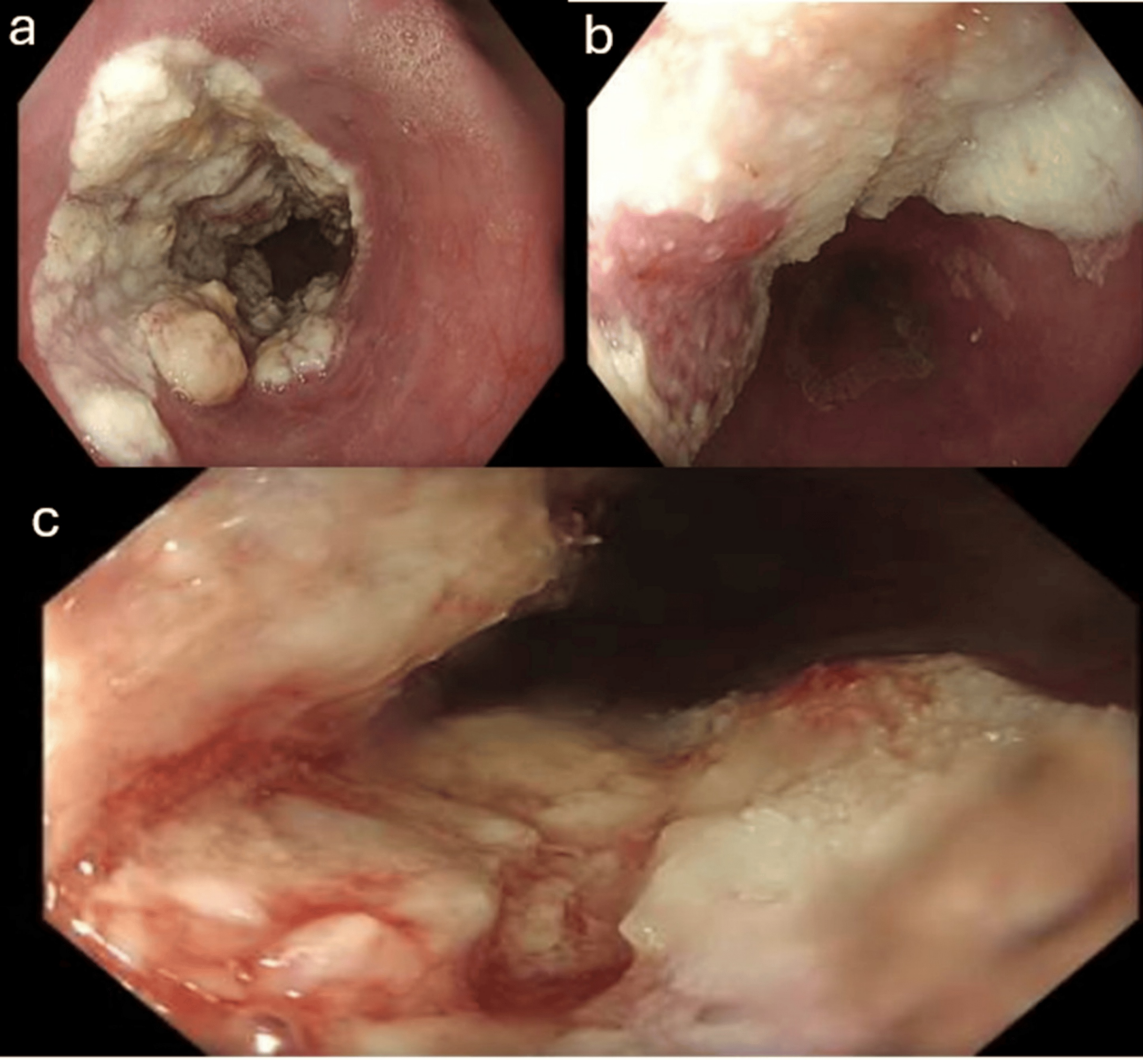
OGD showing esophageal parakeratosis with moderately erythematous mucosa and a plaque in the lower third of the esophagus. (a and b) Esophageal parakeratosis extending from 34 cm to 39 cm from the incisors, with some areas partially circumferential and causing slight luminal narrowing. (c) Moderate erythematous mucosa with a plaque in the lower third of the esophagus. OGD, esophagogastroduodenoscopy

By October 2024, OGD demonstrated moderate erythematous mucosa with plaque in the lower third of the esophagus (Figure 3).

**Figure 4:**
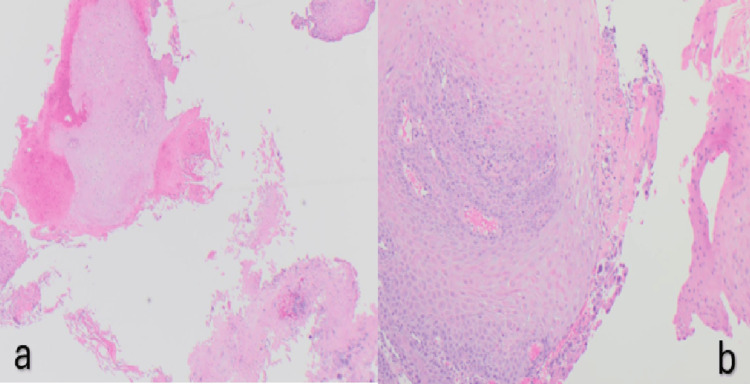
Histopathological slides (a and b) showing prominent esophageal parakeratosis.

Biopsies showed hyperkeratosis/parakeratosis and no fungal or bacterial infection or dysplasia (Figure 4). Supplementation with zinc and riboflavin (vitamin B2) was initiated alongside ongoing acid suppression.

**Figure 5:**
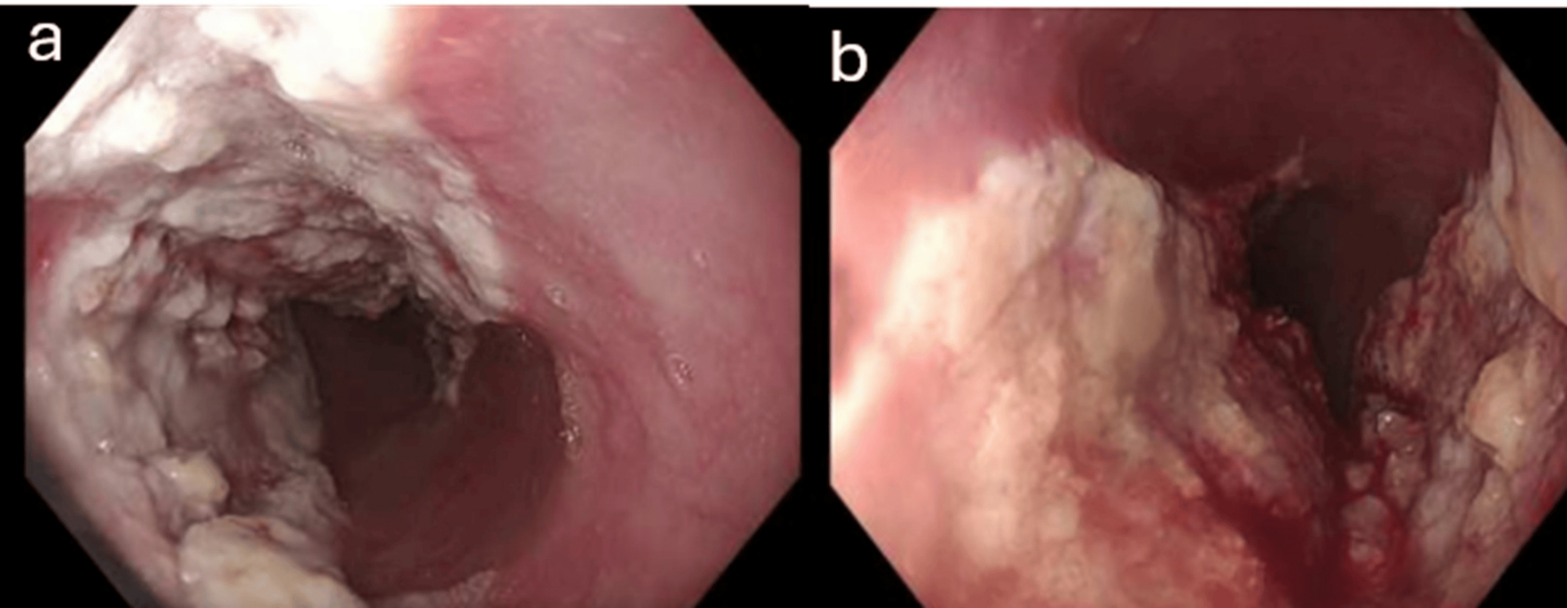
OGD showing parakeratosis spanning roughly 34-40 cm, covering up to about 75% of the circumference. OGD, esophagogastroduodenoscopy

On February 5, 2025, OGD showed the known parakeratosis now spanning roughly 34-40 cm, covering up to ~75% of the circumference but still easily traversable, along with pangastritis; the duodenum was normal (Figure 5).

Histology showed squamous mucosa with persistent parakeratosis in the superficial layer and no evidence of active inflammation, eosinophilic infiltration, intestinal metaplasia, dysplasia, or malignancy. Zinc and vitamin B2 were continued.

**Figure 6:**
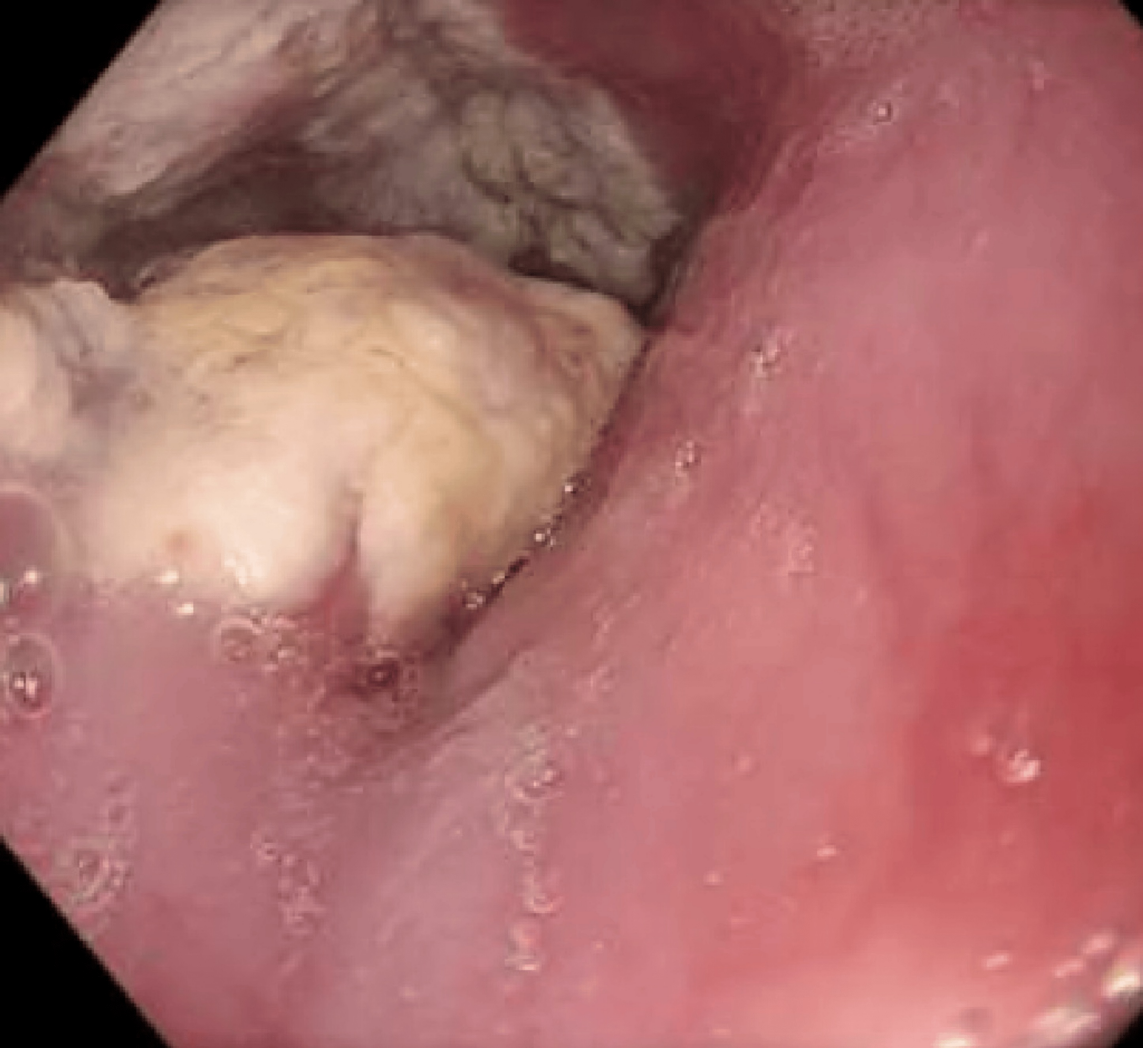
OGD showing parakeratosis (32-38 cm) with easy passage and no fixed stricture. OGD, esophagogastroduodenoscopy

OGD in July 2025 again found the parakeratosis (32-38 cm) with easy passage and no fixed stricture (Figure 6).

Histology identified mild esophagitis and Candida. He received fluconazole 100 mg once daily for 14 days; nighttime famotidine and sucralfate were added to reduce mucosal irritation. Because dysphagia persisted despite the lack of a structural narrowing, esophageal manometry was requested to assess for a motility disorder. Given repeatedly benign histology, a surveillance OGD interval was proposed, with the caveat that earlier reassessment would occur if symptoms evolved.

## Discussion

In our patient, the initial endoscopic appearance of an exophytic, keratinizing, near-circumferential lesion over a long esophageal segment strongly suggested malignancy. However, multiple targeted biopsies consistently revealed hyperkeratosis/parakeratosis without dysplasia or carcinoma. This underscores a critical principle: in suspicious esophageal lesions without a mass on imaging, repeated histologic sampling is essential to avoid premature oncologic intervention.

The linkage of parakeratosis to micronutrient deficiency finds some support in the literature. In a classic animal model, riboflavin deficiency induced marked hyperkeratosis, acanthosis, and pseudocarcinomatous changes in the esophagus, which resolved with riboflavin repletion [3]. In human intervention trials, such as the randomized study by Muñoz et al. in high-risk Chinese populations, supplementation with riboflavin, retinol, and zinc was associated with improvements in markers of esophageal cell integrity, including a reduction in micronuclei frequency; however, these studies did not specifically demonstrate regression of parakeratosis itself [7]. In addition, dietary zinc deficiency in rodent models has been shown to promote proliferative changes in esophageal squamous epithelium [8]. Thus, empiric supplementation with zinc and riboflavin in this patient is biologically plausible, especially given the benign histology, and may contribute to the regression of keratinized mucosa over time. The recurrent identification of Candida on biopsy, despite robust HIV viral suppression and a strong CD4 count, is not unprecedented.

Importantly, the patient was not receiving systemic or inhaled corticosteroids, biologics, or other immunosuppressive medications, and his psoriasis and COPD/asthma did not require therapies that would compromise immunity. Given sustained virological suppression and a strong CD4 count, he remained immunocompetent, making recurrent CE more likely related to local esophageal factors and chronic acid suppression rather than systemic immunosuppression.

In the cohort of immunocompetent patients (no overt immunodeficiency), PPI use emerged as the strongest risk factor associated with CE, with many patients previously treated for reflux [4]. In the Japanese endoscopy series, PPI use was again independently associated with CE (OR 1.69) after controlling for comorbidities [5]. In clinical practice, 63-81% of esophageal candidiasis cases are reported in patients with concurrent PPI use, reflecting a potent pharmacologic association [6]. The mechanistic rationale is plausible: sustained acid suppression may enable Candida overgrowth by altering the esophageal microenvironment, reducing acidity, and disrupting fungal control mechanisms. These findings reinforce the need for careful balancing of PPI use in patients at risk of fungal complications.

In managing CE, standard practice supports systemic azole therapy, commonly fluconazole, for 14-21 days per Infectious Diseases Society of America guidelines; recurrent disease may warrant a suppressive regimen [9]. In people with HIV, drug interactions must be considered, but in our case, moderate-duration fluconazole was feasible. His antiretroviral regimen consisted of tenofovir, emtricitabine, and doravirine, which is a non-boosted combination; therefore, clinically significant drug-drug interactions with azole antifungals such as fluconazole were not anticipated.

Because the patient’s dysphagia persisted without a fixed stricture and the scope traversed easily, an esophageal manometry is planned for the upcoming appointments, as an underlying motility disorder might magnify symptom burden. Surveillance in parakeratosis without dysplasia remains undefined. Given the large segment involved and morphological changes over time, shorter intervals (e.g., 6-12 months) seem safer until stability is documented. Serial endoscopic assessment with generous mapping biopsies remains the best available strategy in the absence of published guidelines.

## Conclusions

Esophageal parakeratosis can convincingly masquerade as malignancy yet represent a benign keratinization disorder, often with potential links to micronutrient deficiency. In people with well-controlled HIV, recurrent CE can still occur, particularly with chronic PPI exposure, and should be managed with guideline-based systemic therapy while minimizing predisposing factors where feasible. This case highlights four practical principles: obtain repeated and generous biopsies before labeling a lesion malignant; consider targeted micronutrient repletion (zinc, riboflavin, and assessment of B vitamins) when parakeratosis is found; rationalize acid suppression and use mucosal protectants to mitigate Candida risk; and individualize surveillance intervals in the absence of formal guidance, with a lower threshold for shorter follow-up when lesions are extensive or morphologically dynamic. Together, these steps minimize overtreatment, prioritize patient safety, and acknowledge the current evidence gaps that warrant future prospective study.
